# Is Philanthropy a Duty?

**Published:** 1891-05-02

**Authors:** Alfred Craske

**Affiliations:** Secretary. North-West London Hospital, Kentish Town Road


### Editor's Letter-Box.

### IS PHILANTHROPY A DUTY?

Sir,—I am extremely gratified to inform you that our treasurer, Mr. George Herring, has received from the Baron de Hirsch de Garenth a donation of £1,000 in aid of the funds of this hospital. You may be interested to know that with the cheque was enclosed a cutting from The Hospital of February 21st, of the article setting forth the urgent needs of the institution, and its claims on the practical sympathy of those "who make philanthropy a duty."—I am, dear Sir, yours faithfully,

Alfred Craske, Secretary.

North-West London Hospital, Kentish Town Road,

